# Computational Nuclear Oncology Toward Precision Radiopharmaceutical Therapies: Ethical, Regulatory, and Socioeconomic Dimensions of Theranostic Digital Twins

**DOI:** 10.2967/jnumed.124.268186

**Published:** 2025-05

**Authors:** Lidia Strigari, Jazmin Schwarz, Tyler Bradshaw, Julia Brosch-Lenz, Geoffrey Currie, Georges El-Fakhri, Abhinav K. Jha, Signe Mežinska, Neeta Pandit-Taskar, Emilie Roncali, Kuangyu Shi, Carlos Uribe, Tahir Yusufaly, Habib Zaidi, Arman Rahmim, Babak Saboury

**Affiliations:** 1 Department of Medical Physics, IRCCS Azienda Ospedaliero-Universitaria di Bologna, Bologna, Italy;; 2 Department of Medical Physics, Memorial Sloan Kettering Cancer Center, New York, New York;; 3 Department of Radiology, University of Wisconsin–Madison, Wisconsin;; 4 Institute of Nuclear Medicine, Glen Burnie, Maryland;; 5 School of Dentistry and Health Sciences, Charles Sturt University, Wagga Wagga, New South Wales, Australia;; 6 Department of Radiology and Biomedical Imaging, Yale University School of Medicine, New Haven, Connecticut;; 7 Department of Biomedical Engineering and Mallinckrodt Institute of Radiology, Washington University, St. Louis, Missouri;; 8 Institute of Clinical and Preventive Medicine, University of Latvia, Riga, Latvia;; 9 Department of Radiology, Memorial Sloan Kettering Cancer Center, New York, New York;; 10 Department of Radiology, Weill Cornell Medical College, New York, New York;; 11 Department of Biomedical Engineering, University of California Davis, Davis, California;; 12 Department of Nuclear Medicine, University of Bern, Bern, Switzerland;; 13 Department of Radiology, University of British Columbia, Vancouver, British Columbia, Canada;; 14 Division of Radiology and Radiological Sciences, Johns Hopkins School of Medicine, Baltimore, Maryland;; 15 Division of Nuclear Medicine and Molecular Imaging, Geneva University Hospital, Geneva, Switzerland;; 16 Department of Nuclear Medicine and Molecular Imaging, University of Groningen, University Medical Center Groningen, Groningen, Netherlands;; 17 Departments of Radiology and Physics, University of British Columbia, Vancouver, British Columbia, Canada; and; 18 United Theranostics, Bethesda, Maryland

**Keywords:** research methods, digital twins, ethical, regulatory, socioeconomic, theranostic

## Abstract

Computational nuclear oncology for precision radiopharmaceutical therapy (RPT) is a new frontier for theranostic treatment personalization. A key strategy relies on the possibility to incorporate clinical, biomarker, image-based, and dosimetric information in theranostic digital twins (TDTs) of patients to move beyond a one-size-fits-all approach. The TDT framework enables treatment optimization by real-time monitoring of the real-world system, simulation of different treatment scenarios, and prediction of resulting treatment outcomes, as well as facilitating collaboration and knowledge sharing among health care professionals adopting a harmonized TDT. To this aim, the major social, ethical, and regulatory challenges related to TDT implementation and adoption have been analyzed. **Methods:** The artificial intelligence–dosimetry working group of the Society of Nuclear Medicine and Molecular Imaging is actively proposing, motivating, and developing the field of computational nuclear oncology, a unified set of scientific principles and mathematic models that describe the hierarchy of etiologic mechanisms involved in RPT dose response. The major social, ethical, and regulatory challenges to realize TDTs have been highlighted from the literature and discussed within the working group, and possible solutions have been identified. **Results:** This technology demands the implementation of advanced computational tools, harmonized and standardized collection of large real-time data, and modeling protocols to enable interoperability across institutions. However, current legislations limit data sharing despite TDTs’ benefiting from such data. Although anonymizing data is often sufficient, ethical concerns may prevent sharing without patient consent. Approaches such as seeking ethical approval, adopting federated learning, and following guidelines can address this issue. Accurate and updated data input is crucial for reliable TDT output. Lack of reimbursement for data processing in treatment planning and verification poses an economic barrier. Ownership of TDTs, especially in interconnected systems, requires clear contracts to allocate liability. Complex contracts may hinder TDT implementation. Robust security measures are necessary to protect against data breaches. Cross-border data sharing complicates risk management without a global framework. **Conclusion:** A mechanism-based TDT framework can guide the community toward personalized dosimetry-driven RPT by facilitating the information exchange necessary to identify robust prognostic or predictive dosimetry and biomarkers. Although the future is bright, we caution that care must be taken to ensure that TDT technology is implemented in a socially responsible manner.

Radiopharmaceutical therapy (RPT) is a rapidly growing oncologic treatment modality rooted in the theranostic ethos of “treat what you see, see what you treat” (1). Diagnostic nuclear medicine imaging, paired with appropriate surrogate therapeutic agents, can potentially help optimize the clinical effectiveness and cost-effectiveness of treatments using patient-specific inputs (2). Unfortunately, current clinical practice related to the choice of RPT agent and injected radioactivities for the most part currently involves a one-size-fits-all activity prescription (3), and there are no established protocols for incorporating patient-specific pharmacokinetics and performing personalized dosimetry-driven treatment planning.

Another article in this series (4) proposes computational nuclear oncology (CNO) as the body of knowledge to truly provide individualized cancer treatment using theranostics for optimized therapy outcome. Theranostic digital twins (TDTs) serve as the platform for CNO, a virtual information construct that is dynamically updated from patient-specific data (5) to personalize and adapt RPT treatment protocols prospectively based on predictive capability to inform decisions (6,7).

TDTs are a subset of biomedical digital twins, which in turn are a subcategory of the more general technology of digital twins. Although a unique objective definition of TDTs is difficult to pinpoint, for our purposes we use the definition given by Boulos and Zhang (8): “A digital twin is a virtual model of a physical entity, with dynamic, bidirectional links between the physical entity and its corresponding twin in the digital domain.” In the case of TDTs, the physical entity is patient-specific anatomy, physiology, and pathophysiology, and the virtual model is the corresponding patient-specific CNO parameterization.

The bidirectional links are provided by nuclear medicine and functional imaging, which guide the parameter estimation and the simultaneous simulation-based optimization of clinical decision-making, including the choice of RPT agent, number of cycles, injection profile, and administered activity per cycle.

Figure 1 illustrates the conceptual framework of the TDT, which incorporates a population-based model with individual patient data, including micro- and macroscopic effects, integrating theranostic imaging, physiologically based pharmacokinetic modeling, and dosimetry using artificial intelligence (AI) tools and mechanistic models to inform decisions and guide precision cancer care, personalized to the individual patient’s needs and circumstances. The realization of such CNO-guided TDTs will require a concerted and collective effort in robust aggregation and integration of heterogeneous data and models across multiinstitutional nuclear medicine cohorts. This, in turn, introduces several practical challenges.

**FIGURE 1.**
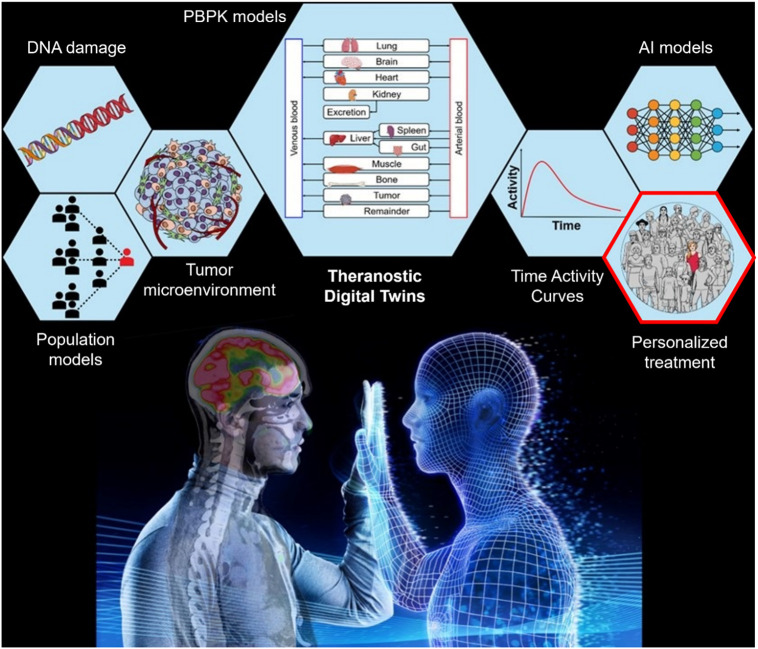
Examples of concepts that use and combine data toward endpoint of personalized treatment. TDT is based on population-based models that are combined with individual patient data such as theranostic imaging-derived time–activity curves. Incorporated micro- and macroscopic effects of treatment are represented by DNA damage, impact of tumor microenvironment, and organ- and tissue-level dosimetry from physiologically based pharmacokinetic (PBPK) modeling. Combination of all these bodies of knowledge on platform of TDT together with AI and mechanistic models will ultimately enable precision cancer care, personalized to individual patient’s needs and circumstances. Realization of such CNO-guided TDTs will require concerted and collective effort in robust aggregation and integration of heterogeneous data and models across multiinstitutional nuclear medicine cohorts. This, in turn, introduces several practical challenges.

The associated paper (not yet published) in this series will touch on some of these, including incomplete knowledge of the CNO etiologic mechanisms, lack of robust and interoperable methods for model integration across scales, and uncertainty on the role of AI methods alongside mechanism-based approaches. In addition to these scientific and technologic challenges, it is necessary to ensure that a TDT adheres to all the specified attributes for its intended purpose, that is, the generation of a digital twin (Table 1).

**TABLE 1.** Key Attributes of Concern for Generation of TDTs

Attribute	Definition
Reliability	Digital twin’s ability to operate consistently and without errors
Robustness	Digital twin’s resilience to unexpected conditions and variations in operating environments
Accuracy	Precision with which digital twin represents its physical counterpart
Usefulness	Degree of benefit and practical value that digital twin provides to users
Validation	Process of ensuring that digital twin meets specified requirements and expectations
Verification	Confirmation that digital twin operates correctly and produces accurate results
Deployment	Process of implementing and putting digital twin into operational use
Integration	Capability of digital twin to work together with other systems and technologies
Scalability	Ability of digital twin to handle increased workloads or to be expanded
Interoperability	Ability of digital twin to interact and communicate effectively with other systems and devices

In TDTs, reliability manifests as the ability to faithfully replicate the behaviors and conditions of the physical system, ensuring consistency and accuracy in its virtual representation. Similarly, reliability is paramount in statistical models to ensure that predictions or estimations are consistently accurate across different datasets. In engineering, reliability extends to the system’s ability to maintain performance under varying operational conditions, safeguarding against failures or deviations from expected behavior. Robustness, another key attribute, underscores the resilience of these models in the face of uncertainty and variability. TDT must be robust enough to handle unexpected changes in the physical environment or operating conditions while maintaining functionality and accuracy. Similarly, statistical models must be robust against outliers or noisy data to provide reliable insights. In engineering, robustness ensures systems can adapt to unforeseen circumstances or disturbances without compromising overall performance.

In addition to these challenges, the TDTs also present several ethical, legal, and social implications that must be accounted for (9,10). To this end, the AI–dosimetry working group of the Society of Nuclear Medicine and Molecular Imaging, focusing on developing the field of CNO for TDTs to personalize RPTs, has identified the key ethical, legal, and social implications as they relate to the TDTs.

## REGULATORY FRAMEWORK FOR AI-BASED TDTS AND RPT

CNO involves AI for multiple applications for the generation of a TDT. Hence, the AI models for TDTs in RPT are influenced by national and international regulations and various legal aspects stemming from different jurisdictions (11–13). The worldwide differences in privacy regulations and the stringency that different regions or jurisdictions impose on the collection, processing, and sharing of personal data can affect how organizations and technologies handle data, especially sensitive health care data, and can present challenges when trying to operate across different geographic zones or comply with multiple sets of regulations (11).

**FIGURE 2.**
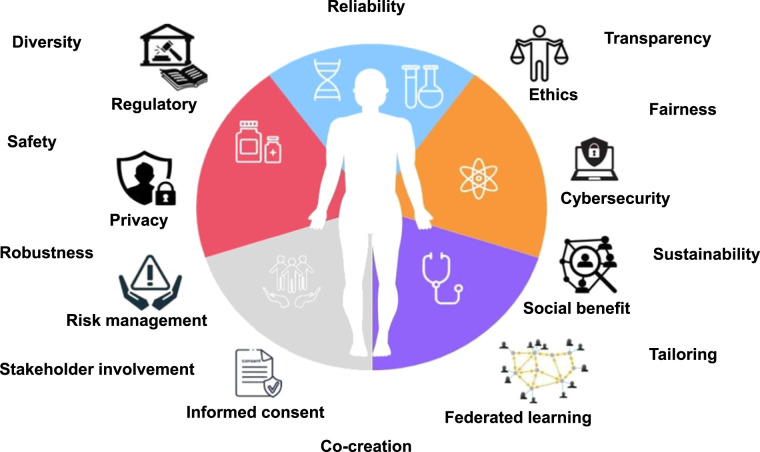
Overview of ethical, regulatory, and socioeconomical dimensions of TDTs.

Figure 2 is a schematic representation of the investigated main issues, whereas Table 2 summarizes for each topic presented in the following paragraphs the key areas of focus and the proposed solutions.

**TABLE 2.** Key Areas of Focus and Proposed Solution for Each Topic Investigated of Regulatory Framework for AI-Based TDTs and RPT

Topic	Key area of focus	Solution
International regulatory dimensions	Diverse data improves AI accuracy and equity	Ensure global cooperation for consistent regulations
Privacy regulation frameworks	Varying privacy laws complicate AI implementation	Address these differences
Federated learning approaches	Federated learning enhances privacy in AI training	Combine federated learning with advanced techniques to develop TDT for RPT and maintain data security
Cross-border data sharing	Unified framework is essential for cross-border data sharing	Streamline compliance to foster global AI development
Ethical and regulatory considerations	Integration of TDTs in RPT presents ethical challenges	Ensure data privacy, consent, bias mitigation, and fairness

### International Regulatory Dimensions

For any medical AI to have a large-scale impact, it is crucial to incorporate source data from different geographic zones to support global health research, personalized medicine, and epidemiologic surveillance (14,15). Such diversity helps capture the complexity of global health care, specifically ethnic factors, variety in health conditions, and treatment responses of different populations, hence allowing AI models to generalize findings, reduce biases, and encourage collaborative innovation. However, the regulatory landscape surrounding the use of AI in health care poses challenges—for example, efforts such as the European Commission’s proposed AI regulatory framework (16) to harmonize issues such as data ownership, consent, and cross-border protection. Global collaboration is vital to create a consistent regulatory environment that balances innovation with patient safety and privacy.

### Privacy Regulatory Frameworks

Differences in privacy regulatory frameworks worldwide pose a major impediment to the seamless operation of AI-based methods for TDTs for RPT. Various laws dictate how health care data can be collected and shared, necessitating careful attention in developing AI-driven solutions that use international datasets (17).

### Federated Learning Approaches

Federated learning offers a strategy to navigate varying privacy regulations by enabling multiinstitutional training without sharing sensitive data. Localized training at different institutions allows insights to be shared while protecting patient privacy by enabling the collective learning necessary for AI-based TDT (18). As federated learning on its own is not a fully privacy-preserving method, it must be combined with formal privacy techniques such as differential privacy or cryptography (18–20) to prevent cyber security attacks from, for example, extracting sensitive data from the AI-based models (21). When applied correctly, federated learning can access substantial data for training AI-based TDTs that would otherwise remain unused because of privacy concerns. The federated learning approaches enable continuous monitoring of TDT performance, though periodic tests and checks are needed to account for the evolution of radiopharmaceuticals and personalization strategies. The TDT longitudinal assessments must consider the evolution of imaging technology, dosimetry, and the availability of refined AI algorithms, similarly to the assessments that radiologists and nuclear medicine physicians must undergo to maintain their accreditation, certification, and credentials. This consideration is valid for each automated system, which needs routine monitoring to detect algorithmic insufficiencies arising from unanticipated interactions with unaddressed inequities, postdeployment changes, or shifts in context and data, particularly when unusual patterns emerge (22).

### Cross-Border Data Sharing

A common global framework is necessary to enable effective data sourcing from various geographic areas, facilitate cross-border data sharing, adhere to privacy regulations, and simplify treatment options for patients with complex risk profiles (23). Streamlining data exchange in this manner would support the global deployment of AI-based methods for TDT and RPT collaboration among international organizations, legal bodies, and health care stakeholders. Such collaborations are essential to strike a balance between innovation, patient privacy, and regulatory compliance in this rapidly evolving field. Current legislation on medical devices (24) increased the requirements for placing new medical devices and keeping existing medical devices on the European Union market, which underscores the need for vigilance and adaptability in this rapidly evolving field.

## ETHICAL AND REGULATORY CONSIDERATIONS IN THE USE OF TDTS FOR RPT

TDTs in RPT are promising, but their application raises crucial ethical concerns, including privacy and security, consent, bias reduction, and fairness in treatment recommendations (25–27). Table 3 summarizes the main topics, key areas of focus, and solutions related to the ethical and regulatory framework of TDTs for RPT, as illustrated in the following paragraphs.

**TABLE 3.** Key Areas of Focus and Proposed Solution for Each Investigated Topic of Ethical and Regulatory Framework for AI-Based TDTs and RPT

Topic	Key area of focus	Solution
Data privacy and security	Adhere to GDPR and HIPAA for patient data protection	Implement encryption, control of access, and regular audits
Cybersecurity	Implement comprehensive cybersecurity measures	Share cybersecurity responsibilities among stakeholders
Informed consent and data sharing	Balance informed consent with ethical data sharing	Use advisory boards and public education to enhance trust
Secondary data analysis	Ensure ethical practices in secondary data analysis	Use techniques such as differential privacy to protect identities
Ethical considerations and implications	Address biases and ensure algorithm transparency	Regularly update TDTs to maintain fairness and relevance in care

HIPAA = Health Insurance Portability and Accountability Act.

### Data Privacy and Security

Ensuring robust data privacy and security is crucial for TDTs in RPT, as they continuously gather sensitive patient data, including imaging and longitudinal clinical measures (e.g., lab values, biomarkers, patient history, and biomarkers). Further, large patient datasets are required for the development of CNO models with or without AI, let alone for the generation of absorbed dose–effect relationship. Therefore, adherence to data protection protocols such as those in the general data protection regulation (GDPR) (28) and the Health Insurance Portability and Accountability Act (29) is critical; these laws mandate rigorous safeguards for patient data. Regular security assessments and audits are integral to promptly identifying and addressing vulnerabilities. Additionally, establishing clear guidelines for data sharing, especially in the context of multiinstitutional collaborations involving TDTs, is essential. This includes delineating responsibilities and protocols for handling patient data, and ensuring all parties adhere to the highest standards of data security. Further, the liability of privacy protection lies clearly within institutions generating the models for TDTs; these institutions can be held accountable for privacy breaches.

### Cybersecurity

Cybersecurity is a critical component in the implementation and operation of TDTs; potential cyber threats can compromise sensitive data and disrupt the evolution and use of TDTs. Comprehensive measures include advanced encryption, rigorous access controls, and continuous monitoring for breaches. The responsibility for cybersecurity lies not just with technical teams but also with health care providers and patients, emphasizing the importance of training, policy development, and awareness.

### Informed Consent and Data Sharing

Balancing informed consent and data sharing with ethical concerns is critical for the development of TDTs for RPT. Researchers must secure informed consent and uphold participant privacy and ethical standards while navigating regulations limiting nonessential data sharing. It would be costly to get explicit informed consent from every data subject for each specific reuse of that subject’s deidentified data. Instead, it is important to notify subjects of the risks associated with the intended reuse and provide them with the opportunity to refuse or withdraw (30), when feasible. When obtaining explicit consent is impossible, information campaigns should be promoted to raise public awareness of the uses of anonymized medical data (31). Multiple notifications to data subjects will better support the trustworthiness of developing TDTs.

### Secondary Data Analysis

TDTs often rely on deidentified data for secondary analysis, enabling them to gain insights from existing datasets. This type of analysis entails a careful and systematic reexamination of deidentified patient data originally collected for other purposes, which may include information such as treatment responses, dosimetry, and patient outcomes. These datasets are rigorously reviewed and repurposed within the DT framework, enabling TDTs to incorporate real-world treatment data and apply advanced analytic techniques to gain insights into the effectiveness of different treatment strategies, optimize dosages, and predict patient outcomes with greater accuracy.

Although these datasets are typically exempt from most human-subject research protections (32) and GDPR requirements when the data are no longer personally identifiable, it is important to acknowledge that although existing regulatory frameworks may be lenient, this does not diminish the ethical duty to establish trust and ensure responsible research practices while using secondary data, for example, absorbed dose distribution, for additional analysis. Researchers must thoughtfully consider factors including subjects’ consent, privacy, and data quality.

In the context of these ethical considerations, specific instances such as the All of Us precision medicine database of the National Institutes of Health highlight the intricacies of balancing privacy with data utility (33). This database has identified a category of data elements that may heighten the risk of unauthorized reidentification while not directly revealing individual participants’ identities, thus underscoring the importance of constraining dataset dimensionality. However, the process of deidentification also carries the potential drawback of diminishing algorithmic CNO model performance. For instance, quasiidentifiers such as patient weight can increase the risk of reidentification but are crucial for calculating SUVs critical for interpreting molecular images and determining absorbed doses. Gauging a feature’s impact on TDT predictability early on can be challenging, but early proof-of-concept projects highlighting vulnerable features with minimal risk for reidentification, for example, weight, make a compelling case for their inclusion in subsequent training datasets. Using differential privacy techniques can help obscure patient-level identifiers while conserving population-level patterns, essential for maintaining model utility in the clinical setting (34).

### Ethical Considerations and Implications

The broader ethical landscape of TDTs in RPT extends beyond the issues discussed thus far. Attention must also be given to the ethical implications in TDT creation, implementation, and application (9,10). Addressing potential biases in the algorithms and ensuring transparency in their development are crucial for algorithm development. Ensuring fairness in treatment recommendations involves preprocessing, inprocessing, and postprocessing strategies. Continuous monitoring and updates of TDTs are necessary to adapt to new evidence and ensure the equitable delivery of care. Further, the models must be trained on diverse datasets to avoid bias and discrimination due to race, cultural background, socioeconomic status, health history, and overall demographics.

Table 4 exemplifies the challenges posed by the GDPR requirements in the exchange of imaging data between Europe and the United States (or other countries).

**TABLE 4.** Challenges Posed by GDPR Requirements in Exchange of Imaging Data Among Europe, United States, and Other Countries

Topic	Key challenge
Definition of personal data	Imaging data and other clinical measures (e.g., clinical features and biomarkers) linked to identifiable individuals (e.g., MRIs with patient metadata) are considered personal data under GDPR, requiring compliance with its regulations
Legal basis for transfer	GDPR mandates specific lawful bases for data processing, especially for exporting and handling processes outside EU; organizations must establish lawful basis for data transfer, such as obtaining explicit consent or using public interest justification, complicating process
Data transfer mechanisms	Transferring data outside EU is permissible only under specific conditions, such as presence of adequacy decision by European Commission that recognizes non-EU country’s data protection regime as equivalent to GDPR; United States does not have such adequacy decision, meaning organizations must rely on mechanisms such as standard contractual clauses or binding corporate rules, which can be complex to implement and manage
Monitoring of compliance	Organizations must ensure ongoing compliance with GDPR, including conducting data protection impact assessments and monitoring data practices, which can be resource-intensive
Potential for data breaches and liability	Discrepancies in regulatory standards raise concerns about data breach liabilities; organizations face heavy fines under GDPR, even for breaches that occur abroad
Cross-border access and subpoenas	U.S. laws may compel organizations to disclose data, potentially conflicting with GDPR obligations, creating challenges for cross-border data sharing
Cultural differences in data privacy	There are differing attitudes toward data privacy between EU and U.S.; European entities may be more cautious about sharing data, impacting trust
Technical and organizational measures	GDPR requires robust technical and organizational measures for data protection, necessitating changes in data handling practices when sharing across borders

EU = European Union.

## RISK MANAGEMENT

Effective risk management in the use of TDTs is critical to ensure patient safety and treatment efficacy. The process begins with identifying and analyzing potential risks, which include technical inaccuracies in CNO models, data privacy breaches, and clinical risks from incorrect treatment recommendations. Mitigation strategies involve rigorous testing and validation of CNO algorithms, use of robust data governance frameworks, and measures to ensure that TDT recommendations are reviewed by health care professionals. Mechanism-based modeling may alleviate potential risks by incorporating the underlying physiologic and mechanistic aspects of the systems being modeled. TDTs should be integrated into clinical workflows cautiously to minimize disruption and enhance adaptability. A key aspect of risk management is prioritizing patient safety, which requires continuous monitoring of TDT outputs and their alignment with patient outcomes. This approach ensures that treatment recommendations are both effective and safe, fostering patient-centric care.

The ongoing process of risk management also involves collaboration with regulatory bodies to remain compliant with evolving guidelines. Continuous improvement is crucial, necessitating regular updates to TDT frameworks based on the latest clinical evidence and technologic advancements. By establishing feedback loops and encouraging collaborative efforts among health care professionals, patients, and regulatory authorities, the use of TDTs in RPT can be optimized, harnessing its potential as a safe and effective tool in personalized medicine.

### Patient Rights and Inclusion

The integration of TDTs in RPT necessitates a steadfast commitment to upholding patient rights and ensuring inclusivity. This commitment is vital to ensure that TDT-based therapies are accessible and beneficial to all patients, irrespective of their backgrounds or demographics. Addressing biases in CNO algorithms is essential to prevent discriminatory treatment recommendations and can be achieved using diverse patient datasets and continually refining TDT frameworks for fair representation of all patient groups, as is our ethical responsibility. Respecting patient rights also involves comprehensive informed consent processes, where patients are fully informed about use of their data and the implications of TDT-guided treatments. Establishing a transparent and inclusive approach in treatment decisions reinforces patient autonomy and trust in the health care system.

Adapting TDTs to different health care settings ensures inclusivity, allowing patients in various geographic and socioeconomic environments access to these advanced therapeutic tools. Engaging patients, health care providers, patient advocacy groups, and policymakers in developing policies that promote broad and equitable access to TDT-guided RPT is crucial. By placing a strong emphasis on patient rights and inclusivity, TDTs in RPT can effectively address the diverse needs of the patient population, leading to more equitable and effective health care outcomes.

### Validation and Clinical Trials

The validation and clinical trial phase is crucial in the development and implementation of TDTs. This phase involves rigorous testing to ensure the accuracy, reliability, and safety of TDTs. Validation includes both algorithmic evaluation, where CNO models are tested against established benchmarks and clinical data, and practical assessments through silent trial testing, where the TDT’s recommendations are compared with actual clinical decisions (35). Clinical trials play a pivotal role, offering insights into the real-world effectiveness and applicability of TDTs. These trials must be conducted with ethical rigor, ensuring patient safety and adherence to regulatory standards. They provide essential data on the efficacy of TDTs in diverse clinical scenarios, helping to refine and improve the technology before its widespread implementation in health care settings. This process not only underscores the scientific validity of TDTs but also builds trust among clinicians and patients in the use of TDTs as a reliable tool in personalized medicine.

### Future Directions and Recommendations

Looking ahead, the field of TDTs in RPT is poised for significant advancements and broader application. Future directions include the integration of more comprehensive and diverse datasets to enhance the accuracy and applicability of AI and CNO models on the platform of TDTs across varied patient populations. Advancements in AI and machine learning will likely lead to more sophisticated and precise TDT frameworks, capable of simulating complex biologic and therapeutic processes with greater fidelity. As technology evolves, continuous reassessment of ethical standards and regulatory frameworks will be necessary to address emerging challenges and ensure patient safety and privacy. Recommendations for the field include fostering collaborative research initiatives, enhancing transparency in TDT development and deployment, and maintaining an ongoing dialogue with regulatory bodies. Emphasis should also be placed on patient education and engagement, ensuring that patients are well informed and actively involved in treatment decisions involving TDTs. By embracing these future directions and adhering to these recommendations, TDTs in RPT can continue to evolve as a transformative tool in personalized health care, improving treatment outcomes and enhancing patient care.

## DATA MANAGEMENT AND INTEROPERABILITY IN TDT DEVELOPMENT

### Data Management Challenges in TDTs

In the realm of CNO, effective data management is crucial for the development and implementation of TDTs. Given the multiinstitutional nature of these initiatives, harmonizing and standardizing data across different sites become essential. This ensures that the data fed into TDTs is consistent, reliable, and conducive to producing accurate, reproducible results.

### Harmonization and Standardization Strategies

Key strategies for data harmonization involve several core approaches. First, implementing standardized imaging protocols across different sites enhances the comparability and reproducibility of imaging data, which are essential for diagnostic accuracy and consistent pretreatment assessments. This approach includes harmonizing parameters such as injected activity, timing between injection and image acquisition, duration of image acquisition, and methods for image reconstruction, alongside system calibration and quality control measures. Second, the use of common data elements in clinical, imaging, and outcome data collection promotes uniformity across sites, making it easier to combine and analyze data from various sources. Additionally, quality control procedures, including image quality assessments and the detection of data outliers, are critical to ensure that data from multiple sites meet established quality standards. Furthermore, adopting interoperable data management systems, which comply with standards such as Digital Imaging and Communications in Medicine, supports smooth data sharing and collaborative research. Finally, data normalization techniques, such as *z* score normalization or normalization to a reference standard, are applied to improve data comparability by reducing site-specific variations.

### Integration with Ethical, Regulatory, and Socioeconomic Considerations

These data management strategies are intrinsically linked to broader ethical, regulatory, and socioeconomic considerations. Standardized data practices can facilitate regulatory compliance and address privacy concerns. Meanwhile, federated learning aligns with ethical standards by enhancing patient data privacy and contributes to the socioeconomic aspect by enabling broader participation and representation in TDT development.

### Challenges and Future Directions

Implementing these strategies is not without challenges, including coordinating across institutions, overcoming technical barriers to interoperability, and ensuring adherence to diverse regulatory requirements. Future directions could focus on developing more sophisticated data harmonization tools and expanding the scope of federated learning to encompass a broader range of data types and sources, further enriching the TDTs in precision RPT.

## SOCIAL CHALLENGES AND OPPORTUNITIES FOR AI-BASED TDT AND RPT

### Social and Clinical Added Value of TDT: Encouragement of Clinical Acceptance

Researchers not only bear responsibility to data subjects but also bear the responsibility of demonstrating the social and clinical value of their research. For TDTs to make effective decisions, they must be regularly updated with evolving datasets, ensuring accuracy, completeness, and timeliness. The TDT’s clinical performance, fairness, and trustworthiness depend significantly on the quality of the dataset used for training, making it crucial to avoid data pollution. Defining data features and establishing a quality assurance process before data collection are essential to mitigate this risk, as these factors greatly influence TDT performance.

The development of TDTs must focus on interpretability and explainability to facilitate shared decision-making between patients and clinicians. Techniques such as saliency maps and parallel models are used to elucidate the inputs and uncertainty levels in predictions. However, deploying these techniques requires caution. The varying explanation needs for different contexts are often conflated, and it remains uncertain whether clinicians should delineate every decision-making pathway. Current techniques may not provide reliable individual-level information, which challenges the concept of explainability that presupposes an individualized computational understanding of patient-level outcomes. Although explainability and interpretability techniques are technically intriguing, their deployment in clinical settings should be approached with caution. They may not consistently yield accurate individual-level predictions and could potentially hinder clinicians’ ability to identify incorrect outputs, leading to automation bias. Thus, whereas these techniques hold technical interest, they are not yet ready for clinical application and require further research. In the interim, ensuring transparency about the intended uses and accuracy of TDTs might be adequate for clinicians to adequately inform patients about the risks and benefits.

### Socioeconomic (Logistic and Economic Barriers)

A significant economic barrier to long-term clinical implementation and development of TDTs in RPTs is the current lack of reimbursement for sequential quantitative imaging and data processing to perform treatment planning and posttreatment verification of the absorbed doses. Other barriers include a lack of scanners, technologists, and physicists to scan and perform dosimetry for the exponentially increasing number of patients receiving ^177^Lu-labeled RPTs (36).

### Bias and Risk Management in TDT Development for Health Care

Addressing potential bias in social categories such as race, sex, and nationality is crucial because of their intricate relationship with patient-level variables. AI solutions, such as generative adversarial networks and variational autoencoders, are effective in generating synthetic data to balance and regularize databases (37). Generative adversarial networks, with their dual-network structure, and variational autoencoders, which discern and replicate data structures, help counter data imbalances or inadequacies, thereby enhancing AI model accuracy (38). Key questions include determining liability for decisions made using TDTs and ensuring interoperability within complex, multilayered TDT systems. Developers are responsible for creating safe, effective, and beneficial TDTs across diverse clinical contexts. This stems from the ethical principles of nonmaleficence and beneficence. Achieving safety and efficacy requires task-specific designs involving domain experts, high-quality datasets, and aligned performance metrics. Concurrently, TDTs must be generalizable to various clinical settings, including low-resource environments and diverse patient populations. Although demanding extensive effort and data, specification of limitations for particular populations, imaging equipment, or tasks is essential for trustworthy TDTs. Early identification and intervention are necessary to mitigate additional risks. Establishing flexible and adaptable contracts with stakeholders can reduce barriers to TDT implementation, accommodating the evolving nature of the technology. Automated systems in patient care must be regularly monitored to identify any algorithmic discrimination due to unforeseen interactions with unaddressed inequities, postdeployment changes, or shifts in patient data and context. Providers should assess these systems to prevent discriminatory outcomes, conducting evaluations routinely and whenever unusual patient care patterns arise (39).

### Interoperability

Interoperability is essential in TDTs for RPT, facilitating the effective exchange and use of information between physical and virtual representations of patients within the TDT. This includes seamlessly incorporating diverse treatment inputs such as chemotherapy, immunotherapy, and organ reserve data into the TDT framework. This integration allows TDTs to interact in real time with different therapeutic modalities, harnessing data to optimize treatment plans, improve decision-making, and personalize patient care.

### Development of Guidelines and Recommendations

There is a pressing need to develop comprehensive guidelines and recommendations for end users and stakeholders, including decision-makers, patients, and citizens, on constructing TDTs for RPT. These guidelines should focus on using temporal activity distributions, such as those derived from physiologically based pharmacokinetic models, and provide clear directives on how to effectively store and share these models, including their inter- and intraorgan relationships. It is imperative that scientific associations responsible for developing these guidelines also identify potential risks associated with TDTs and propose strategies for their mitigation. This will ensure that TDTs are constructed, used, and shared to maximize their clinical value while minimizing potential risks to patients and health care systems.

### Incorporation of Patient Involvement or Autonomy

Developers should identify strategies to favor the incorporation of patient value (40) and autonomy into the TDT. These strategies comprise the development, for patients, of a graphical user interface with which health care professionals can explain TDT predictions and added values.

### Cocreation Process of TDT

The cocreation process of TDT can enhance ethical and legal aspects by engaging patients directly, ensuring that their perspectives and concerns are addressed, which promotes informed consent and autonomy. Collaborative development fosters transparency, building trust and facilitating regulatory compliance. Tailoring digital twin solutions to patients’ needs will improve treatment effectiveness while adhering to legal standards. Ongoing stakeholder involvement allows for continuous assessment and adjustment of ethical and legal practices. Developing shared ethical and legal guidelines ensures standardized and compliant practices. Addressing disparities in access and benefits promotes equity and justice, ensuring fair distribution of technology and health care advantages. Additionally, educating stakeholders on ethical and legal issues creates a culture of responsibility and awareness.

## CONCLUSION

We have presented ethical and regulatory aspects and concerns for the generation of TDTs as promising tools for improving RPTs. However, whereas technologic advancements promise significant improvements in simulation and data analysis, it is crucial to consider the ethical, legal, and social implications. Data security and privacy remain critical challenges, whereas reliance on accurate data necessitates rigorous management and verification of sources. Patient empowerment and personalized care are positive outcomes, but ethical issues regarding equitable access to technologies and sensitive data management must be addressed. We highlighted potential barriers to TDT development and implementation and identified solutions to leverage TDTs for optimized RPTs. Close collaboration among technologic, clinical, ethical, and legal stakeholders is essential to maximize the benefits of TDTs, mitigate associated risks, and ensure a positive impact on health and society.

## DISCLOSURE

Arman Rahmim and Carlos Uribe are cofounders of Ascinta Technologies Inc. No other potential conflict of interest relevant to this article was reported.

## Notes

KEY POINTS **QUESTION:** How do the ethical, legal, and social implications of digital twin technology in nuclear medicine theranostics impact patient care and data management? **PERTINENT FINDINGS:** The integration of digital twins in nuclear medicine theranostics significantly improved the accuracy of personalized treatment approaches. However, substantial concerns regarding data security, equitable access, and longitudinal assessment of its performance were identified. **IMPLICATIONS FOR PATIENT CARE:** Digital twin technology can enhance personalized care in nuclear medicine theranostics, addressing data privacy, ensuring equitable access, and ensuring the cocreation process. Including all the stakeholders could be crucial for early uptake of TDT in RPT, maximizing patient benefits and maintaining ethical standards.
